# Advances in T Helper 17 Cell Biology: Pathogenic Role and Potential Therapy in Multiple Sclerosis

**DOI:** 10.1155/2015/475158

**Published:** 2015-12-07

**Authors:** Elisabetta Volpe, Luca Battistini, Giovanna Borsellino

**Affiliations:** Neuroimmunology Unit, Santa Lucia Foundation, Via del Fosso di Fiorano 64-65, 00143 Rome, Italy

## Abstract

The discovery of the T helper (Th) 17 lineage, involved in the protection against fungal and extracellular bacterial infections, has profoundly revolutionized our current understanding of T cell-mediated responses in autoimmune diseases, including multiple sclerosis (MS). Indeed, recent data demonstrate the pathogenic role of Th17 cells in autoimmune disorders. In particular, studies in MS and in its animal model (EAE, experimental autoimmune encephalomyelitis) have revealed a crucial role of Th17 cells in the pathogenesis of autoimmune demyelinating diseases in both mice and humans. Over the past years, several important aspects concerning Th17 cells have been elucidated, such as the factors which promote or inhibit their differentiation and the effector cytokines which mediate their responses. The identification of the features endowing Th17 cells with high pathogenicity in MS is of particular interest, and discoveries in Th17 cell biology and function could lead to the design of new strategies aimed at modulating the immune response in MS. Here, we will discuss recent advances in this field, with particular focus on the mechanisms conferring pathogenicity in MS and their potential modulation.

## 1. Introduction

Differentiation of naive CD4^+^ T cells into T helper (Th) cells with diverse effector functions is crucial for the establishment of an adaptive immune response. Until recently, only two major cell subsets, Th1 and Th2, were used to describe the different adaptive immune responses established to eradicate pathogens [1–3]. Th1 cells induce cell mediated inflammatory responses against intracellular bacteria [4–7], while Th2 cells activate a protective response against helminth infection [8]. However, persistent or uncontrolled effector T cell responses are also associated with pathological states and tissue damage: an excessive Th2 cell response is responsible for atopic diseases, such as asthma [9], and an abnormal Th1 cell response can mediate chronic inflammation and is involved in several autoimmune diseases [10, 11]. In 1998 the discovery of CD4^+^ T cells producing IL-17 [12] unveiled the presence of another subset of Th cells, the Th17 subset, distinct from Th1 and Th2 [13, 14], and its discovery has helped the understanding of immune responses unexplained by the Th1/Th2 paradigm, such as the response against fungi like* Candida albicans* [15] and extracellular bacteria such as* Pseudomonas aeruginosa* [16],* Klebsiella pneumoniae* [17],* Streptococcus pneumoniae* [18], and* Staphylococcus aureus* [19], and the development of autoimmune disorders, such as multiple sclerosis (MS), Crohn's disease, psoriasis, and rheumatoid arthritis. The pathogenic role of Th17 cells in autoimmune diseases is supported by both human studies and experiments performed in animal models. Indeed, IL-17A is highly expressed in the central nervous system (CNS) lesions and in the blood and cerebrospinal fluid (CSF) of patients with MS [20–24], in the colonic mucosa of patients with ulcerative colitis or Crohn's disease [25, 26], in the psoriatic skin [27, 28], and in the synovial tissues from rheumatoid arthritis patients [29]. Studies in murine models such as experimental autoimmune encephalomyelitis (EAE) [30], trinitrobenzene sulfuric acid- (TNBS-) induced colitis [31], and antigen or collagen-induced arthritis [32] reveal that the IL-17 pathway plays a pathogenic role in autoimmune disorders. Finally, the concept that Th17 cells are responsible for driving autoimmune inflammation was finally established when EAE, the mouse model of MS, was shown to be induced by passive transfer of IL-17-producing myelin reactive CD4 T cells [33].

In this review we discuss our current understanding of the Th17 lineage, focusing on the factors regulating their differentiation, their typical features, their pathological roles in MS, and the potential modulation of their response for therapeutic approaches.

## 2. Cytokine Production by Th17 Cells

IL-17 is the cytokine produced specifically by Th17 cells. IL-17A (commonly referred to as IL-17) is part of a cytokine family including IL-17B, IL-17C, IL-17D, IL-17E (also known as IL-25), and IL-17F [34]. All members of the family show some conserved regions: IL-17A and IL-17F (the only cytokines of this family produced by Th17 cells) are the most similar to a 55% homology and exert similar functions [35]; IL-25 has the sequence with lowest similarity to IL-17A (only 16%) and plays distinct roles in immunity, mainly regulating the Th2 response against helminthic parasites and allergic inflammation [36–38]. IL-17B, IL-17C, and IL-17D have been shown to induce the production of proinflammatory cytokines, but their biological function is largely unknown [39–42]. Recent studies by three different groups have highlighted the function of IL-17C in mucosal immunity and in autoimmune responses [43–45].

Within the IL-17 family of cytokines, the biological function and regulation of IL-17A and IL-17F are the best understood. Both are produced by Th17 cells and can also act as heterodimers [46]. The effective signalling of IL-17A and IL-17F requires the IL-17 receptor (IL-17R), a heteromeric complex consisting of IL-17RA and IL-17RC [47]. Although both receptors are extensively expressed in different tissues and cell types [48–50] functional studies have focused mainly on epithelial cells. Both IL-17A and IL-17F induce epithelial cells to produce granulopoietic colony stimulating factor (G-CSF), stem cell factors that regulate granulopoiesis, and CXC chemokines (CXCL1, CXCL2, CXCL5, and CXCL8) responsible for neutrophil recruitment [51–53]. IL-17A increases the expression of mucins such as MUC5AC and MUC5B in primary human bronchial epithelial cells* in vitro* [54]. In addition, IL-17A also induces the expression of human beta defensin-2 [55] and CCL20 in lung epithelial cells [56]. This cooperative induction of neutrophil recruitment and antimicrobial-peptide production improves epithelial-barrier integrity and may be critical for mucosal host defense against extracellular bacteria and fungi.

Moreover, several studies have documented the role of IL-17 in regulating antibody generation by plasma cells and germinal center and ectopic inducible bronchus-associated lymphoid tissue (iBALT) formation [57–59].

Altogether, these functions of IL-17 make Th17 cells potentially relevant for vaccine development: in experimental models, Th17 cells are effective in providing vaccination-induced immunity against a range of pathogens, and the identification of Th17-specific antigens for common prevalent pathogens could help formulate a serotype-independent effective vaccination strategy [60].

**Figure 1:**
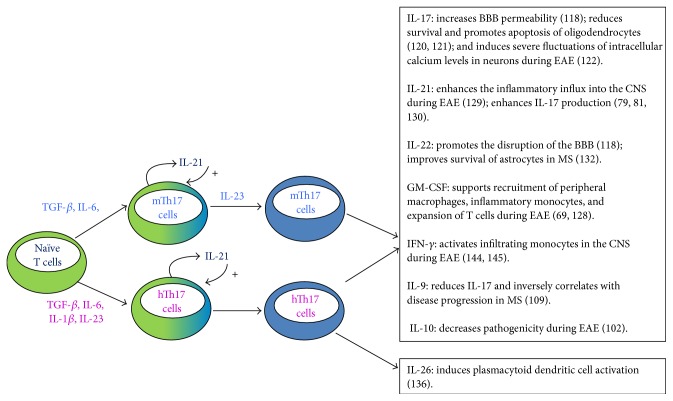
Differentiation of Th17 cells and their potential functions in MS. The T helper (Th) 17 cell differentiation program in mice (blue labels) and humans (purple labels) shares significant similarities. In both organisms, IL-6, TGF-*β*, IL-23, and IL-21 are involved in complete Th17 differentiation. Human Th17 cell differentiation requires also IL-1*β*. Fully differentiated Th17 cells produce specific sets of cytokines. Both murine and human Th17 cells produce IL-17, IL-21, IL-22, GM-CSF, IFN-*γ*, IL-9, and IL-10, with potentially relevant functions in MS pathogenesis. Human Th17 cells also produce IL-26.

However, the Th17 cytokine profile is not restricted to IL-17 production. In fact, Th17 cells are potentially producers of a broad array of cytokines, including IL-21, IL-22, IL-26, IL-6, TNF, and in certain conditions also GM-CSF, IL-9, IL-10, and IFN-*γ* [61–70]. Because each of these cytokines has different functions, they collectively affect the global outcome of the Th17 response and generate different Th17 responses (Figure 1).

## 3. Stimuli Required for Th17 Cell Differentiation

Human* in vitro* studies reveal that dendritic cells (DCs) exposed to bacteria or fungi, but not viruses, elicit strong Th17 responses [62, 80]. Among pathogen-associated molecular patterns (PAMPs), peptidoglycan (TLR2 agonist) [62, 80] and its product muramyl dipeptide (NOD2 agonist) [80] are the most potent stimuli for the production of IL-17. In mice, an alternative pattern-recognition pathway activated by fungal infection has also been described, involving the engagement of a C-type lectin receptor, dectin-1, by fungal *β*-glucans components of zymosan [81]. Moreover, DCs stimulated with the pure *β*-glucan curdlan (dectin-1 agonist) prime T cells for a much higher IL-17 production than does IFN-*γ* [82].

Stimulation of DC by pathogen-derived structures induces the production of cytokines which ultimately drive Th17 cell differentiation [83, 84]; these cytokines have been identified by using antigen presenting cell- (APC-) free models of T cell polarization. In mice, several reports have shown that transforming growth factor-*β* (TGF-*β*) and IL-6 [85–87] have a critical role in inducing Th17 differentiation. Although not necessary, other proinflammatory cytokines such as IL-1*β*, TNF, and IL-21 can enhance this differentiation [87, 88]. Finally, IL-23 induces expansion of murine Th17 cells both* in vitro* [87] and* in vivo* [89] (Figure 1).

Studies on human Th17 cell differentiation have reported a critical role for IL-1*β*, IL-6, IL-23, and TGF-*β* [65, 66]. TGF-*β* was also shown to act in synergy with the inflammatory mediator IL-21 in driving Th17 cell differentiation [90]. Moreover, in humans it has been demonstrated that supernatants from DCs stimulated with zymosan or *β*-glucan induce the development of Th17 cells with requirements for TGF-*β*, IL-1*β*, and IL-6 [91]. Collectively, these studies reveal that similar pathways regulate both human and mouse Th17 cell differentiation [92] (Figure 1).

## 4. Transcription Factors Required for Th17 Cell Differentiation

The key transcription factor involved in the differentiation program of Th17 cells is the retinoic acid-related orphan receptor (ROR) *γ*t [93, 94], a member of the ROR family of nuclear receptors encoded by the* RORC* gene. Studies in mice [93] and humans [65, 66] revealed that ROR*γ*t controls the expression of IL-17A and IL-17F by Th17 cells. However, ROR*γ*t does not regulate all genes related to the Th17 lineage, suggesting that other transcription factors contribute to the expression of genes involved in its functional differentiation.

Although all cytokine pathways involved in Th17 cell differentiation result in the upregulation of ROR*γ*t expression, IL-6, IL-21, and IL-23 signalling pathways additionally activate STAT3, which directly binds the IL-17 and IL-21 promoters [95, 96]. Cells transduced with both ROR*γ*t and an active form of STAT3 (STAT3C) produce more IL-17 per cell, suggesting a cooperation between ROR*γ*t and STAT3 at transcriptional target sites [63]. Patients with autosomal-dominant hyper-IgE syndrome associated with dominant-negative mutations in* STAT3* lack Th17 cells, revealing the importance of STAT3-dependent signals in the differentiation and/or expansion of human IL-17-producing cells [97].

IRF4 has also been reported to be essential for Th17 cell differentiation [98]. IRF4-deficient mice have an impaired ROR*γ*t induction, since IRF4 is located upstream of ROR*γ*t in the Th17 cell differentiation process [98].

Another transcription factor known to cooperate with ROR*γ*t is the basic leucine zipper transcription factor ATF like (BATF), which is upregulated in Th cells following T cell receptor (TCR) activation [99, 100] and which is essential for the maintenance of ROR*γ*t expression induced by TGF-*β* and IL-6 [100]. Recent studies have identified IRF4 and BATF as “pioneer factors” that bind and govern the accessibility of chromatin, enabling ROR*γ*t recruitment and binding to Th17 signature genes [101, 102].

Another member of the ROR family, ROR*α*, also contributes to mouse Th17 cell development, and coexpression of ROR*α* and ROR*γ*t causes synergistic increases in IL-17A, IL-17F, and IL-23R expression [103]. ROR*α* and ROR*γ*t bind to the same retinoid response-like elements individually or as heterodimers and may be functionally redundant [104].

Retinoic acid receptors can be regulated by the ligand-dependent transcription factor aryl hydrocarbon receptor (AHR) [105, 106], whose binding to environmental pollutants such as dioxin influence Th17 cell differentiation [107, 108]. Different cellular contexts might provide distinct transcriptional partners for AHR and determine diverse outcomes of the immune response.

Another transcription factor associated with environmental conditions is the hypoxia-inducible factor 1*α* (HIF1*α*), which is a key sensor of hypoxia. HIF1*α* directly binds and drives transcription of RORC [109], and lack of HIF1*α* results in diminished Th17 development [110].

Therefore, combinatorial interactions of multiple transcription factors, including ROR*γ*t, ROR*α*, activated STAT3, IRF4, BATF, AHR, HIF1*α*, and other unidentified factors regulate the genes that define the Th17 lineage. Decoding this transcriptional network will provide a better understanding of pathways involved in the differentiation of Th17 cells and may facilitate the development of strategies to manipulate the immune responses associated with these cells.

## 5. Immunomodulation of Th17 Responses

The conjunct action of multiple soluble factors fine-tunes and regulates the outcome of immune responses. For instance, IL-10 has an important immunomodulatory role during the Th17 response. In fact, IL-10 production by restimulated mouse Th17 cells in the presence of TGF-*β* and IL-6 is able to regulate Th17 cell immunopathology and reduces EAE disease severity [111]. In contrast, restimulation in the presence of IL-23 does not induce IL-10 production, conferring pathogenic functions to Th17 cells [111]. Thus, IL-10 production in Th17-polarizing conditions may be a mechanism of self-regulation of the potentially dangerous Th17 cell response. In humans, the regulation of IL-10 production in Th17 conditions is dependent on IL-1*β* [65, 112]. Human Th17 cell differentiation in the presence of IL-1*β* results in an enhancement of IL-17 and in a decrease of IL-10 production suggesting that, during the resolution of inflammation, a decrease in or a lack of IL-1*β* may simultaneously decrease the production of IL-17 and enhance the production of IL-10 [65, 112]. Moreover, several studies suggest that IL-10 inhibits differentiation of Th17 cells in a direct manner [113, 114]. Given the role of IL-10 as a potent negative regulator of inflammation [115, 116], its presence could be an important mechanism for controlling the Th17 response.

The cytokine IL-2 has a similar inhibitory effect on Th17 cell differentiation. In fact, addition of exogenous IL-2 reduces the proportion of murine Th17 cells differentiated from naive T cells in the presence of TGF-*β* and IL-6, whereas inversely blocking autocrine IL-2 by the addition of neutralizing antibodies enhances Th17 differentiation. Furthermore, IL-2-deficient mice contain a substantially greater fraction of Th17 T cells, and* in vitro* stimulation of T cells from these mice results in higher proportions of IL-17-producing cells [117]. In contrast, IL-2 seems to have a positive effect on IL-17 expression by human Th17 cells, and the addition of an IL-2-blocking antibody during differentiation prevents cell proliferation and IL-17 production [66].

We recently demonstrated that IL-9 has an inhibitory effect on IL-17 production by human Th17-polarized cells. Importantly, the interaction between IL-17 and IL-9 reveals a decisive mechanism regulating the pathogenic inflammation generated by Th17 cells in MS [118]. In fact, we found that IL-9 level in the CSF of MS patients inversely correlates with the progression of MS and with the levels of IL-17 observed in the CSF, indicating that inhibition of IL-17 via IL-9 could be protective in MS [118], despite the controversial results on the role of IL-9 in EAE [68, 119].

Th17 responses can also be suppressed by antigen presenting cells producing IL-27: this cytokine inhibits IL-17 production by human and mouse Th17 cells [120]. Interestingly, plasma levels of IL-27 negatively correlate with the percentage of circulating Th17 or with plasma IL-17 concentration in patients with progressive MS, suggesting that IL-27 might be involved in this disease [121].

Thus, the establishment, progression, and outcome of chronic inflammation, which underlies the pathogenesis of MS, are highly dependent on the nature of the complex network of cytokines which modulate Th17 cells and which are produced during the immune response.

## 6. Pathogenic Role of Th17 Cells in Multiple Sclerosis

MS is a heterogenous disease characterized by a wide variety of neurological symptoms and signs attributed to discrete areas of inflammation, demyelination, and axonal loss in the CNS [122]. Two main courses of MS exist: relapsing-remitting (RR) and primary or secondary progressive (PP and SP). RR-MS is characterized by recurrent neurologic symptoms interspersed by periods of stability, with full or partial recovery; the progressive form is characterized by gradual neurological dysfunction with or without exacerbations [123, 124].

Immunological mechanisms such as myelin destruction by specific CD8 T cells, activated microglia, invading macrophages, natural killer cells, and autoantibodies produced by B cells contribute to demyelination and axonal loss. Importantly, Th17 cells are mainly responsible for the persistent inflammation that characterizes both forms of MS [122].

Among the typical features of Th17 cells that could confer pathogenicity to MS, their abilities to enter the encephalic compartment, to penetrate the blood brain barrier (BBB), and to recruit inflammatory cells have been documented. In particular, Th17 cells express high levels of the C-C chemokine receptor 6 (CCR6) on the cell surface [62, 125] that binds the C-C chemokine ligand 20 (CCL20) constitutively expressed by the vascular endothelium of the blood-cerebrospinal barrier, thus enabling the entry of Th17 cells into the encephalic compartment through the choroid plexus [125]. Once in the brain parenchyma, Th17 cells release several proinflammatory mediators including IL-17A, responsible for the downregulation of tight junction proteins of the BBB, increasing BBB permeability, and favoring migration of both soluble inflammatory molecules and other circulating immune cells into the CNS [126]. Consistent with murine studies, IL-17A levels in the CSF of MS patients are associated with neutrophil expansion and blood brain barrier disruption, indicating that IL-17 may have similar pathogenic roles in EAE and MS [127].

It has been proposed that IL-17 also interferes with remyelinating processes, reducing survival [128], and promoting apoptosis of oligodendrocytes, the myelin-forming cells [129]. Moreover, a potential neurotoxic effect of Th17 cells has also been reported, by the induction of severe fluctuations of intracellular calcium levels in neurons during EAE [130] or through the release of granzyme B in human fetal neurons [126].

However, murine studies suggest that not only production of IL-17, but also other features of Th17 cells collectively confer encephalitogenic potential to these cells in MS. In fact, the course of EAE is unperturbed or only lightly ameliorated in IL-17 knockout mice and in wild-type mice treated with neutralizing antibodies specific for IL-17A [131, 132].

Among the proinflammatory mediators produced by Th17 cells, GM-CSF is gaining much attention in MS pathogenesis. Recent studies have suggested that this cytokine plays a fundamental role in the pathogenicity of Th17 cells in EAE [69, 70]. In fact, GM-CSF-deficient mice are resistant to EAE [69, 133] and GM-CSF production by Th17 cells is crucial for their capacity to induce EAE [70, 134], likely supporting recruitment of peripheral macrophages, inflammatory monocytes, and expansion of T cells [69, 135, 136].

Another cytokine produced by Th17 cells is IL-21, whose administration before induction of EAE enhances the inflammatory influx into the CNS as well as the severity of the disease [137], likely due to the role of IL-21 in the induction and expansion of Th17 cells [88, 90, 138]. Interestingly, IL-21 and its receptor have been detected in lymphocytes infiltrating acute and chronic active white matter MS lesions [139], underlining the role for this cytokine in CNS inflammation.

The role of IL-22 in MS is still unclear. High levels of IL-22 and of IL-22 producing cells have been detected in the serum, CSF, and peripheral blood of MS patients [140–142], and it has been demonstrated that IL-22 promotes the disruption of BBB* in vitro* and* in vivo* [126]. However, in the brain tissue IL-22 improves survival of astrocytes that express its receptor [140], thus suggesting a protective role of this cytokine in MS.

Among the cytokines produced by Th17 cells, IL-26 has never been studied in the context of brain inflammation, due to the fact that only human Th17 cells produce it, thus making the EAE model inappropriate. However, the identification of a risk locus containing* IL26* and single-nucleotide polymorphisms within the* IL26* gene region associated with MS [143] encourages the study of its role in MS. Interestingly, IL-26 forms complexes with bacterial DNA and self-DNA released by dying cells, thus inducing plasmacytoid dendritic cells to produce IFN-*α* [144], that could have important implications in the modulation of the autoimmune response.

Moreover, the potential pathogenic role of Th17 cells in MS and in other autoimmune disorders could be related to their enhanced capability to survive, self-renew, generate effector progeny, and enter the memory pool with an efficiency superior to that of Th1 cells [145], and to their resistance to activation induced cell death [146–148]. In particular, we have recently demonstrated that human Th17 cells derived from healthy donors and MS patients express lower levels of FASL compared to Th1 cells, with a consequent lower sensitivity to cell death [148]. This mechanism could explain the persistence of IL-17-producing cells in autoimmune diseases, such as MS, contributing to the chronic inflammation typical of the disease.

Interestingly, Th17 cells could play a pathogenic role in MS also by converting their phenotype into a proinflammatory Th1 profile, as demonstrated by IL-17F reporter mice, where committed Th17 cells give rise to a progeny that shifts toward enhanced IFN-*γ* expression, contingent upon limited or absent TGF-*β* [149].

Similarly, in humans there are evidences indicating that, in the presence of IL-12, Th17 cells produce also IFN-*γ*. These cells which produce both IL-17 and IFN-*γ*, called Th1/17 cells [26], together with “nonclassical Th1 cells” might contribute to disease pathogenesis through properties shared by both the Th1 and Th17 subsets [150]. Moreover, Th17 cells producing IFN-*γ* are enriched in myelin oligodendrocyte glycoprotein-specific T cells [151]. IFN-*γ* produced by these cells could strongly activate macrophages [152] whose infiltration in the CNS correlates with EAE severity [153].

## 7. Therapeutic Approaches Targeting Th17 Cells in MS

In recent years several therapies directed against Th17-related cytokines, including IL-17, IL-23, and GM-CSF, have been developed, and some are currently being tested in ongoing clinical trials (Table 1). The fully humanized antibody neutralizing IL-17A called AIN457 or Secukinumab (NCT01708603 Clinicaltrial.gov) is already approved for the first-line systemic treatment of moderate to severe plaque psoriasis [154, 155]; in MS patients, although it showed a reduction by 63% of new magnetic resonance imaging (MRI) lesions compared to placebo-treated patients, the reduction of annualized relapse rate (ARR) was not statistically significant [71]. Moreover, Secukinumab was ineffective and resulted in higher rates of adverse events, mainly infections, compared with placebo in patients with Crohn's disease [156], and the study in MS terminated early based upon development of another anti-IL-17 monoclonal antibody with better potential for treating MS patients, Ixekizumab (NCT02387801 Clinicaltrial.gov).

Another Th17-related cytokine that has recently gained attention, as a promising molecular target, is GM-CSF, and MOR103 (NCT01517282 Clinicaltrial.gov) a fully human monoclonal antibody against human GM-CSF was tested in clinical trials. However, although performed on a limited number of patients, the first clinical trial did not show efficacy in MS [73].

Selective targeting of single Th17 cytokines, such as IL-17 or GM-CSF, with monoclonal antibodies has not shown efficacy in MS and in other autoimmune diseases such as Crohn's disease and rheumatoid arthritis, although the involvement of Th17 cells in their pathogenesis has been widely documented. This suggests that Th17 cells' pathogenicity relies also on other factors, and thus targeting this T cell subset, inhibiting the Th17 differentiation program as a whole, may be more effective in limiting inflammation.

In this context, Ustekinumab, an antibody neutralizing the p40 subunit common to IL-12 and IL-23, was developed but the clinical trial has been completed and did not show efficacy in reducing inflammation in MS (NCT00207727 Clinicaltrial.gov) [74–76]. However, by blocking both IL-12 and IL-23 which sit upstream of the Th1 and Th17 differentiation program, respectively, this monoclonal antibody suppressed both of these responses and this could explain its low efficacy in MS, considering that interference with Th1 responses determines increased susceptibility rather than protection from EAE [157–159]. Specific inhibitors of the p19 subunit of IL-23, including Tildrakizumab, Guselkumab, AMG 139, BI 655066, and LY3074828, that have been developed and are being currently tested in other autoimmune diseases [72] could give encouraging results in MS treatment.

The ROR*γ*t transcription factor represents the ideal target for the manipulation of Th17 cell responses. Digoxin, a small molecule which binds ROR*γ*t and interferes with transcription, has been shown to inhibit Th17 cell differentiation in the mouse and to reduce EAE severity [77], and another natural product, ursolic acid, has similar effects [78]. Chemical modification of other small molecules shown to bind the ligand-binding domains of ROR*α* and ROR*γ*t has led to the development of SR1001, which reduces human and murine Th17 cell differentiation, and suppresses the clinical severity of autoimmune disease in mice [79]. Although still in preclinical phase, the results of these studies indicate that this novel class of compounds has potential utility in the treatment of autoimmune diseases.

## 8. Conclusions

Over the past few years, remarkable advances in the understanding of Th responses have been reported. The discovery of the Th17 subset, of the cytokines and transcriptional factors regulating its differentiation, and of the biological functions of its effector cytokines has advanced our understanding of the role of CD4^+^ T cells in adaptive immunity. However, many issues remain to be addressed, especially concerning the balance between pathological and protective roles during autoimmune and infectious diseases. Advances in these points are critical for the future development of new therapeutic strategies able to modulate Th17 pathways for the treatment of MS and of diseases where Th17 cells play a pathogenic role.

## Figures and Tables

**Table 1:** Therapeutic approaches targeting Th17 cells in MS.

Agent	Functional role	Clinical trial identifier in MS	Clinical stage	Reference
Secukinumab (AIN457)	Neutralizes IL-17A	NCT01708603	63% reduction of new MRI lesions compared to placebo; ARR reduction not statistically significant	[71]

Ixekizumab	Neutralizes IL-17		Currently tested in psoriasis	[72]

MOR103	Neutralizes GM-CSF	NCT01517282	Did not show efficacy in MS	[73]

Ustekinumab	Neutralizes the p40 subunit common to IL-12 and IL-23	NCT00207727	Did not show efficacy in MS	[74–76]

Tildrakizumab	Neutralizes the p19 subunit specific of IL-23		Currently tested in other autoimmune diseases	[72]

Guselkumab	Neutralizes the p19 subunit specific of IL-23		Currently tested in other autoimmune diseases	[72]

AMG 139	Neutralizes the p19 subunit specific of IL-23		Currently tested in other autoimmune diseases	[72]

BI 655066	Neutralizes the p19 subunit specific of IL-23		Currently tested in other autoimmune diseases	[72]

LY3074828	Neutralizes the p19 subunit specific of IL-23		Currently tested in other autoimmune diseases	[72]

Digoxin	Interferes with ROR*γ*t		Preclinical phase	[77]

Ursolic acid	Interferes with ROR*γ*t		Preclinical phase	[78]

SR1001	Interferes with ROR*α* and ROR*γ*t		Preclinical phase	[79]
